# Estimated Dietary Intakes of Toxic Elements from Four Staple Foods in Najran City, Saudi Arabia

**DOI:** 10.3390/ijerph14121575

**Published:** 2017-12-14

**Authors:** Hatem Mohamed, Parvez I. Haris, Eid I. Brima

**Affiliations:** 1 Faculty of Health and Life Science, De Montfort University, Leicester LE1 9BH, UK; hatem.mhamed@hotmail.com (H.M.); pharis@dmu.ac.uk (P.I.H.); 2 Department of Chemistry, College of Science, King Khalid University, Abha 61413, Saudi Arabia

**Keywords:** toxic, elements, rice, wheat, meat, chicken, risk assessment, EDI, Najran, KSA

## Abstract

Exposure of the inhabitants of Najran area in Saudi Arabia to the toxic elements As, Cd, Cr, and Pb through foods has not been previously investigated. Exposure to such elements is an important public health issue, so the study described here was performed with the aim of determining estimated dietary intakes (EDIs) for these metals in Najran area. The As, Cd, Cr, and Pb concentrations in four staple foods (rice, wheat, red meat, and chicken) were determined by inductively coupled plasma-mass spectrometry. A food frequency questionnaire (FFQ) was completed by 80 study participants. These data were used to estimate dietary intakes of the metals in the four staple foods. The mean As, Cd, Cr, and Pb EDIs in the four food types were 1.1 × 10^−6^–2.6 × 10^−5^, 1.42 × 10^−5^–2.2 × 10^−4^, 3.4 × 10^−4^–8.0 × 10^−4^, and 2.3 × 10^−5^–2.1 × 10^−3^ mg/kg bw day, respectively. Hazard Quotients (HQ) for all elements did not exceed one. The highest Pb concentration was found for chicken and the source of this toxic element in this food needs to be investigated in the future. The lowest As concentration was found for wheat highest in rice. The EDIs for all elements in the four food types were below the provisional tolerable weekly intakes set by the World Health Organization (WHO).

## 1. Introduction

Toxic elements are normally found in foodstuffs with various concentrations [1]. The concentrations of toxic elements in foods depend on the type of food and the route through which the food was exposed to the toxic element. These elements can enter foodstuffs from the environment in different pathways such as contaminated water with untreated municipal, industrial waste and polluted air [2]. Food consumption is considered to be the main pathway, the other pathways being inhalation, dermal contact contaminated water, through which humans are exposed to potentially toxic elements [3,4].

In the Kingdom of Saudi Arabia (KSA) the use of treated water (wastewater) is considered as a main alternative supply for crops in the agricultural sector that can be beneficial to soil; however, the disadvantages of using wastewater in irrigation system in agriculture sector pose a potential health risks [5]. The accumulation of toxic elements including heavy metals such as Cd, Cu, Fe, Mn, Pb and Zn in foods can originate from both geogenic as well as anthropogenic sources [6]. Heavy metals can accumulate in water at toxic levels. For example, As is found at relatively high concentrations in groundwater in certain parts of the world and can contaminate vegetables and cultivated grains such as rice [7]. Consumption of large amounts of food contaminated with heavy metals may lead to adverse health effects, such as neurological disorders, cancer, damage to important body organs, such as the kidneys, lungs and liver [8,9]. Exposure to heavy metals should always be below the guideline recommended by the World Health Organization WHO [10].

Rice is one of the main staple foods worldwide. Rice grain is rich in a number of bioactive compounds including ferulic acid, phytosterols and oryzanol, but can also contain heavy metals such as Cd, Hg, and Pb [11,12]. Also, wheat is a major source of various vitamins, metals, nutrients, dietary fiber, and supplies the human body with considerable amounts of fat [13,14]. Heavy metals such Pb, Cd, Zn, Cr and Ni that exist in soil environment with high concentrations may affect nutrient content and enzyme activities of wheat [15]. Red meat is very rich in protein containing all the essential amino acids, vitamins and minerals (including vitamins B6 and B12, P, Se, and Zn) that are required for tissue formation, growth and repair [16]. However, some red meat and meat products may contain relatively high concentrations of toxic elements and supply heavy metals to the human body [17]. Chicken meat is considered as an excellent source of protein, phosphorus, zinc and iron [18]. High intakes of chicken contaminated with heavy metals may pose serious risks to human health [19].

The behaviors and toxicities of heavy metals are still poorly understood, and little information is available on their toxicities, specifically in the KSA. Currently, there are no specific guidelines for regulating the concentrations of different metals in KSA, although the establishment of the Saudi Food and Drug Authority is likely to address these issues. The Gulf Cooperation Council (GCC), which is a regional regulatory body that is known as GCC Standardization Organization (GSO) is also playing a role in the area of food safety [20]. Very little information is available regarding the concentration of toxic elements in foods consumed in Saudi Arabia, which relies heavily on imported foods from various countries around the world.

This is the first study to investigate the presence of toxic elements in some staple foods, sold in local markets that are commonly consumed in Najran KSA. The aim of this study was to determine the estimated dietary intakes (EDIs) for the toxic elements As, Cd, Cr, and Pb in the main staple foods (rice, wheat, red meat, and chicken) of the inhabitants of Najran city, Saudi Arabia. The findings will be valuables for providing food safety information as well as dietary guidance for protecting the health of the population residing in this region and other parts of KSA.

## 2. Materials and Methods

### 2.1. Location

**Figure 1:**
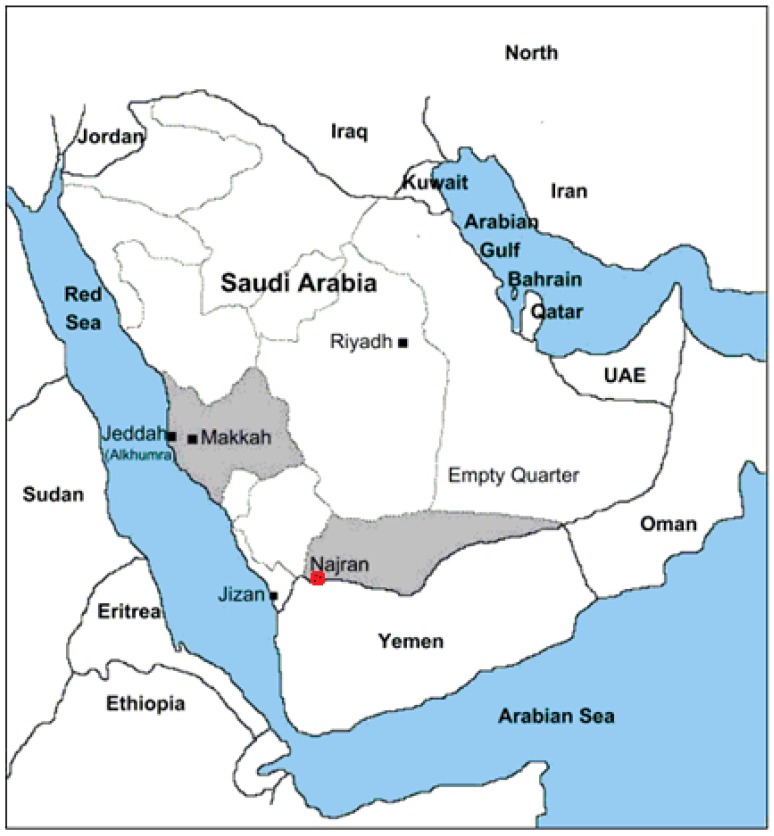
Location of studied area (modified from Google maps).

The study location was in Najran city (Figure 1) in Najran province in the south of KSA. The food samples were collected from Najran city, and analysed by using ICP-MS in Abha city at King Khalid University.

### 2.2. Population

Demographic parameters (gender, age, marital status, occupation and educational status) and dietary intake were collected by using a Food Frequency Questionnaire (FFQ). A modified form of the FFQ was used based on a previous study [21]. Each participant older than 18 years was included in this study. The participants were consisted of 35 males and 45 females. Moreover, informed consent was obtained from each participant. Ethical approvals were obtained from both Ethic committees at De Montfort University and Najran University.

### 2.3. Food Sample Collection

Different food categories were included, and specific attention was paid to staple foods rice, wheat, red meat and chicken. The questions in the FFQ were designed to obtain quantitative and qualitative information about food consumption patterns, specifically focusing on the staple foods of the Najran population. Questions were asked about portion sizes (in grams) of the different foods and consumption frequencies. The final amount of each food consumed was calculated by multiplying the portion size by the consumption frequency and then scaling the results to a unit of one day.

The food samples in this study were collected based on local market availability. In total 25 samples of a representative number of the four main staple foods (rice = 8, red meat = 4, wheat = 5 and chicken = 8) belonging to the most popular commercial brands or manufacturers were purchased from different food shops or from the same store in El-Faisaliya market of Najran city, Saudi Arabia. Food samples products and brands were collected in July 2017.

The collected wheat samples were from Tawaheen Al Ayrisa-Helbah Yemeni, Tawaheen Al Ayrisa-Samah Najran, Tawaheen Al Ayrisa-Kialegee Dubai, Tawaheen Al Ayrisa-Zeraae Najran, Matahen & Mahamas and Al Jazeera-Smrah Najran. Rice samples collected were as follows: Chopstick golden rice-American Rice, Abu Kass-Basmati Rice, Perfumed brown rice-Gaba Rice, Al Walimah-Basmati Rice, Indian Gate-Basmati Rice, Rice King-Basmati Rice White, Al Shealan-Basmati Rice, Mahtma brown rice-Natural and Whole Grain Rice. Red meat samples collected were Beef-shoulder-Al Athyabeh Butcher, Camel-Al Athyabeh Butcher, Goat-shoulder-Al Feysaleh Butcher and Lamb-shoulder-Al Khaledeh Butcher. Chicken samples were BBQ chicken-Al Makbos Restaurant, Compressed chicken-Al Makbos Restaurant, Grilled chicken-Al Faysiliah Restaurant, Grilled chicken-Al Airseh Restaurant, BBQ chicken-Al Reemal Restaurant, Compressed chicken-Al Reemal Restaurant, Shawarma chicken-Al Airseh Restaurant and Shawarma chicken-Al Faysiliah Restaurant.

### 2.4. Pre-Treatment of Food Samples

All rice and wheat samples were grounded and the powder was stored in plastic bags until they were digested. All the other samples were washed with deionized water, and then dried overnight at 80 °C in an oven for moisture calculation purpose. The dried samples were grounded to powder and kept in plastic bags until they were digested. The samples treatment method was based on a method previously published by Al-Rmalli [22].

### 2.5. Digestion of Food Samples

Approximately 0.5 g of a powdered dried food sample was mixed with 4 mL of 70% HNO_3_ (Sigma-Aldrich, St. Louis, MO, USA) and 2 mL of H_2_O_2_ (Sigma-Aldrich), and the mixture was digested for 40 min using a Multiwave 3000 microwave sample preparation system (Anton-Paar, Graz, Austria). The digestion method was based on a recent method published by Brima [23].

### 2.6. Elemental Analysis

All glassware were washed with dilute HNO_3_, using deionised water (>18 Ω/cm) and rinsed with the deionized. An iCAP Q inductively coupled plasma mass spectrometer (Thermo Fisher Scientific, Waltham, MA, USA) was used to determine As, Cd, Cr, and Pb concentrations in the digested sample solutions. The iCAPQ operating conditions were: RF power 1550 W; cool gas flow rate 14.1 L/min; nebuliser gas flow rate 0.94 L/min; auxiliary gas flow rate 0.79 L/min; dwell time 0.01 s; total time for each measurement 3 min. A stock solution containing the elements of interest was prepared from a 10.0 ± 0.05 μg/mL mixed standard (ULTRA Scientific, North Kingstown, RI, USA). An internal standard solution containing Sc was prepared from a 1.0 g/L solution (ULTRA Scientific). The stock solutions were prepared in 1.0% HNO_3_.

### 2.7. Analytical Method

The analysis was performed in the same way as in a recent study [23]. A series of multi-element calibration standards containing As, Cd, Cr, and Pb at concentrations in the range of 0.0 to 40.0 μg/L were used. The internal standard solution (scandium) at a concentration of 100 μg/L was introduced online. A quality control mixed standard of a 20 μg/L containing the elements of interest was analysed after each group of five samples. The data were processed using Qtegra software (Thermo Fisher, Waltham, MA, USA).

### 2.8. Health Risk Assessment for Humans and Estimated Dietary Intake Calculation

Concentrations and EDIs were calculated for each element in the four staple foods based on recent publication [24]. The daily intake of each measured element in food was calculated based on the following equation:

DI (µg) = D (g) × Cc (µg/g)(1)

where DI = daily intake; D (g) = D is the average daily intake in all the food types (in g/day) and Cc = Calculated concentration (µg/g).

EDIs were expressed in mg/kg bw day which is the human risk assessment and was calculated as in the following equation:

EDI = (DI (µg)/kg bw)/1000 = mg/kg bw day(2)

where BW = is the average body weight (in kg), average body weight in this study is 71.53 kg based on the participated volunteers.

Hazard quotients (HQs) for As, Cd, Cr, and Pb in all 25 samples were calculated based on the following equation.

HQ = EDIcalc/EDIRf(3)

In Equation (3), EDIcalc is the EDI found in this study and EDIRf is the EDI (PMTDI) established by the WHO [25,26,27,28].

## 3. Results

### 3.1. Quality Control

Quality control was performed by measuring known concentration after each five measurements. The concentration of each element (20 µg/L) was spiked into test samples, and the recoveries were all higher than 90% in the four food samples as shown in Table 1. Concentrations of the investigated elements in the 25 food samples analysed are presented in Table 2.

### 3.2. Average Intake of the Four Staple Foods

**Figure 2:**
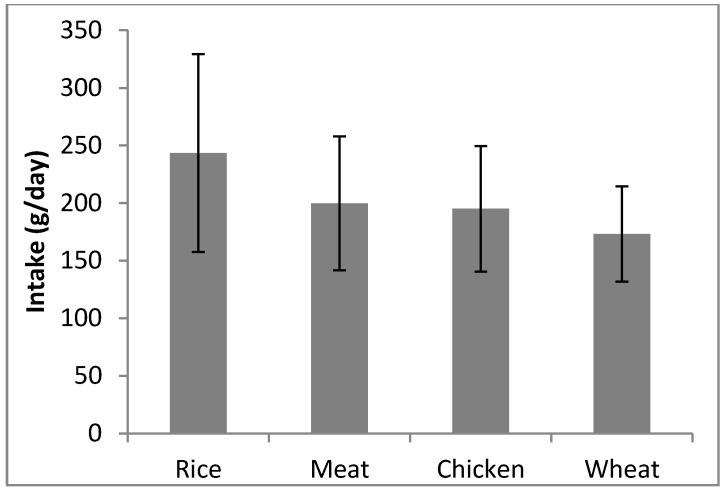
Average daily intakes of staple foods by group of population in Najran city.

The average daily intakes of different foods by the study participants are shown in Figure 2. These were based on data collected from 80 people residing in Najran who completed a FFQ. The participants were all over 18 years and consisted X females and y males. The average daily intakes of rice (243 ± 86.0 g) was highest. The average daily intakes of the other foods decreased in the order: red meat (199.8 ± 58.1 g) > chicken (170.4 ± 42.3 g) > wheat (161 ± 58.3 g).

### 3.3. Concentration of As, Cd, Pb and Cr in the Four Staple Foods

The mean As, Cd, Cr, and Pb concentrations (µg/g) in the 25 samples of rice, wheat, red meat, and chicken are presented in Table 2. The average as concentrations were lower than the concentrations of the other elements analysed in all the samples. The average concentration of Pb in the different foods was the highest while Cd was the lowest.

### 3.4. Daily Intakes of Toxic Elements

The average daily intakes (µg/day) of As, Cd, Cr, and Pb in rice, wheat, red meat, and chicken for the inhabitants of Najran are shown in Table 3.

The daily intakes of As, Cd, Cr, and Pb from rice, wheat, red meat, and chicken varies widely, as shown in Table 3. The highest daily intake of As comes from rice (8.18 µg/day) followed by red meat (1.86 µg/day). Chicken contributes to the highest intake of Cd (15.37 µg/day). The intake of Pb is very high from chicken (149.79 µg/day), and lowest from red meat (1.62 µg/day). The highest daily intake of Cr is from rice (56.93 µg/day).

### 3.5. Estimated Provisional Tolerable Weekly Intakes (PTWIs) and Provisional Maximum Tolerable Daily Intake (PMTDI)

Table 4 shows the estimated provisional tolerable weekly intakes and provisional maximum tolerable daily intake taken from the published literature.

**Figure 3:**
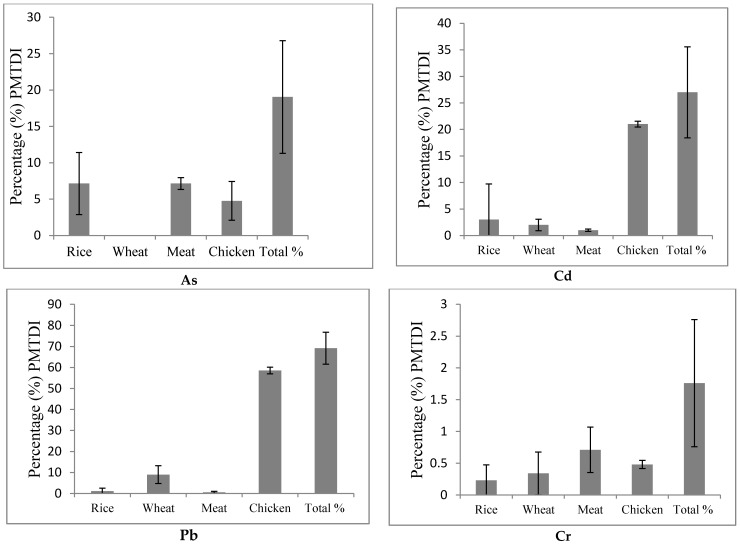
Percentage (%) PMTDI of As, Cd, Pb and Cr from each food class, and all food classes combined, that were investigated in the current study.

Figure 3 illustrates the provisional maximum tolerable daily intake (PMTDI) contributions from each food class and all food classes combined-for each element; As, Cd, Pb and Cr. The %PMTDI for Pb is much higher compared to the other elements. Chicken contributes the highest %PMTDI for Pb compared to the other foods. Red Meat and rice contribute the highest percentage PMTDI for As. The %PMTDI is less than 100% for each foods and is also below 100% when the four foods are combined.

Table 5 shows the estimated dietary intake of toxic elements and their hazard quotient in the four main staple foods for As, Cd, Cr, and Pb. As can be seen, the HQ for each of the elements does not exceed 1. The highest HQ is for Pb in chicken (0.59) and As in rice (0.27).

## 4. Discussion

This is the first study to estimate the dietary intakes of As, Cd, Pb and Cr from main four staple foods (rice, red meat, wheat and chicken) that are commonly consumed in a population from Najran city, KSA. The toxic elements composition of these foods are discussed in the context of public health implications for this population. Food containing toxic elements can pose risks to human health, depending on the toxic elements concentrations in the food and the amount of the food consumed. Kuhnlein et al. described the concept of tolerable intakes that is widely used as the tolerable daily intake or tolerable weekly intake, to describe safe levels of toxic elements [29]. The PTWI developed by the Joint Food and Agriculture Organization/WHO Expert Committee on Food additives is defined as the maximum amount of a contaminant a person can be exposed to per week over a lifetime without posing unacceptable risks to health [30].

### 4.1. Concentrations of Heavy Metals in Main Four Foods

#### 4.1.1. Concentrations of Heavy Metals in Rice

In this study the As, Cd, Cr, and Pb concentrations in rice samples were found to be less than the WHO recommended limits [30]. Rice is a potentially major source of As in humans [31,32,33]. There is a great deal of published evidence that some types of rice contain high concentrations of both organic and inorganic As and can pose risks to human health [32,33]. The concentration of As seen in the basmati rice samples collected from markets in Najran are lower than the range reported previously [33]. Our results are lower than the results of a study in China in which the highest As concentration in rice was found to be 0.96 mg/kg [34]. It was also lower than the findings of another study with distribution range of 0.08–0.20 mg/kg [35]. A study in Bangladesh had found that As concentrations were highest in short broad rice (0.06–0.96 mg/kg, mean 0.36 mg/kg) and decreased with increasing grain size, being 0.07–0.33 mg/kg (mean 0.17 mg/kg) in medium slender rice and 0.03–0.30 mg/kg (mean 0.16 mg/kg) in long slender rice [36]. High As concentrations pose health risks that are manifested in almost every part of the human body [37]. In this study, we found that the hazard quotient for As from rice intake is far lower than 1. However, it needs to be stressed that we only analysed eight rice samples and therefore more screening of As concentration in rice consumed in KSA is needed, including the speciation of As, in order to establish a comprehensive risk assessment [32,33].

#### 4.1.2. Concentrations of Heavy Metals in Wheat

This study indicates that the concentrations of toxic elements investigated in all wheat samples did not exceed the WHO guidelines. However, some previous studies, from other parts of the world, reported high concentrations of heavy metals in wheat samples. For instance, wheat irrigated with contaminated wastewater and grown in China compared with clean irrigation water contained As, Cd, Cr, and Pb concentrations of 18.2, 3.8, 326.7 mg/kg, and 6.6 mg/kg, respectively, more than three times the WHO guidelines. The results of that study suggest heavy metals can accumulate in wheat and can potentially pose health risks to people consuming wheat grown at sites irrigated with contaminated wastewater [38]. In our study, the HQs for As, Cd, Cr, and Pb in all wheat samples were all <1.0, as was also found for a range of toxic elements in a similar study in Argentina [39]. It appears from the current study that wheat consumed in Najran do not contain very high concentrations of toxic elements. However, we only analysed few samples and regular monitoring should be done to ensure that consumed wheat do not contain unsafe levels of toxic elements.

#### 4.1.3. Concentrations of Heavy Metals in Meat

The As, Cd, Pb, and Cr concentrations in all red meat samples were low, at 0.01, 0.01, 0.01 mg/kg, and 0.25 mg/kg, respectively. Our results are similar to the results of a study performed in Spain, in which low As, Cd, and Pb concentrations (2.71 ± 0.27, 7.88 ± 0.80 µg/kg, and 6.45 ± 1.14 µg/kg, respectively), were found in red meat [40]. Also, our findings agree with the results of a study performed in Taiwan, that had found low concentrations of As (0.029 ± 0.006 mg/kg), Cd (0.451 ± 0.102 mg/kg), Pb (0.168 ± 0.276 mg/kg) and Cr (0.837 ± 0.071 mg/kg) were found in meat [41]. However, our results disagree with the findings of a study carried out in Algeria in red meat samples that revealed high concentrations of some toxic metals Cd, Cr, and Pb were 0.132–2.165 µg/g, 0.768–2.335 µg/g, and 0.260–1.411 µg/g, respectively. These values were higher than the guidelines limits set by the local health authorities [42]. Also, in another study carried out in Lahore, Pakistan, it was found that the concentrations of Pb (0–4 ppm) in meat samples exceeded the permissible limits set by international organizations [43]. Red meat containing heavy metals at concentrations that exceed the PTWIs recommended by the WHO can be harmful to health [44]. In this study, the HQs of As, Cd, Cr, Pb in red meat samples were lower than one. Accordingly, it can be assumed that consuming red meat is safe for the inhabitants of Najran. Our results agreed with the results of a study performed in Enugu, Nigeria, in which the hazard quotients were lower than 1, suggesting that people in Enugu are not exposed to significant health risks by consuming red meat [45].

#### 4.1.4. Concentrations of Heavy Metals in Chicken

The results of this study show that the average concentrations of As, Cd, Cr, and Pb in chicken samples were 0.01 mg/kg, 0.06 mg/kg, 0.62 mg/kg, and 0.14 mg/kg, respectively. The concentrations levels of all these metals were found to be within the permissible limits. However, the concentration of Pb in chicken was found to be higher compared to the other elements. As a consequence the percentage PMTDI for Pb was found to be rather high and when Pb intake from all the other foods and beverages are considered it could exceed the PMTDI. Therefore, it is recommended that the food safety authorities monitor Pb levels in chicken and identify the source of Pb in chicken meat. This is important since the consumption of chicken is very high in KSA. In a previous study from Brazil, chicken meat was found to be safe for human consumption because the daily intakes of nonessential elements such as As (10.5 ± 7.2a (4.5–28.8 ng/g)), Cd (1.1 ± 0.8a (0.4–2.6 ng/g)), and Pb (7.3 ± 4.5a (1.8–17.4 ng/g)) in chicken meat were below toxic levels [46]. On the other hand, there are other studies in the literature, including one from southern Nigeria, in which some heavy metals Cd (0.01–5.68 mg/kg^−1^); Cr (0.01–3.43 mg/kg^−1^), and Pb (0.01–4.60 mg/kg^−1^) were found in chicken meat at higher concentrations above the permissible limits [47]. Also, high Pb (0.01–0.40 µg/g) concentrations, exceeding the recommended limit for human consumption, have been found in chicken in Turkey [48]. Our findings were in agreement with the study findings in Valdivia, Chile, that had found the daily intakes of As (0.13 µg/g) in chicken were found to be within the safe limits and pose no health risks [49]. High heavy metals concentrations can be found in chicken meat because chickens can be exposed to heavy metals in their feeds. [50]. The As, Cd, Cr, Pb and HQs found in our chicken samples were all within the appropriate guidelines. Our results agrees with the result of a study performed in Ghana, in which the hazard quotient for all the toxic elements that were analysed in chicken samples were less than one, indicating that exposure to toxic metal from chicken meat could be significant [51].

### 4.2. Toxic Elements Estimated Deitary Intake (EDI)

Table 5 showed the estimated dietary intake and hazard quotient for the toxic elements (As, Cd, Pb and Cr) in all the 25 samples. Our study results showed the range of EDI (mg/kg bw day) of the toxic elements (As 1.11 × 10^−6^–1.14 × 10^−4^ mg/kg bw day), (Cd 1.42 × 10^−5^–2.15 × 10^−4^ mg/kg bw day), (Pb 1.31 × 10^−4^–2.09 × 10^−3^ mg/kg bw day) and (Cr 7.96 × 10^−4^–4.76 × 10^−4^ mg/kg bw day) in rice, wheat, red meat and chicken, respectively. The HQ was found to be less than 1 for all the foods investigated and therefore the consumption of these foods do not pose health risks for the Najran population. However, it is important to point out that we only analysed four classes of foods (rice, wheat, chicken and meat) and more studies are necessary to establish if the HQ for each of the toxic elements will still remain below 1, when other foods and beverages are included in the risk calculations. The HQ for Pb from chicken was 0.59 and it is possible that exposure to Pb could be a cause for concern for the KSA population when the intake of this toxic element from other foods and beverages are considered. Our results differ from the findings of a study in Bangladesh where the consumption of main staple foods had shown that the estimated dietary intake of some heavy metals [Pb (4.92 mg/kg bw day), Cr (6.99 mg/kg bw day), Cd (8.44 mg/kg bw day) and As (8.35 mg/kg bw day)] were higher [52]. A previous study carried out in India found that the heavy metals in grains were found to pose no health risks [53]. Likewise, the EDIs of some heavy metals, including As, Cd, and Cr, in cereals and other foods in study in Kermanshah City, western Iran, were found lower than the PTWIs and within the ranges reported for similar cereals from various parts of the world [54]. A study in the Region Lagunera, Mexico in had found that the EDI of As (0.94 and 0.76 mg/kg bw day) was lower than the recommend PTDI by WHO [55]. In a study performed in Riyadh City, Saudi Arabia, on some representative food items in the Saudi diet, it gave an Pb EDI of 22.7 and 24.5 µg/person day that are lower than the PTDI recommend by The Joint Expert Committee on Food Additives (JECFA) [56]. As yet a total diet study (TDS) investigating the dietary intake of toxic elements for KSA has not been carried out. Therefore, more work needs to be done to monitor EDI in major foods consumed by people in Najran city as well as other cities within KSA and the public health authorities should advise people to diversify their diet and consume healthy food.

## 5. Conclusions

The concentrations of As, Cd, Cr, and Pb in four staple food samples; rice, red meat, wheat and chicken were analysed. Higher Pb concentration was seen in chicken, while As concentration was lowest in wheat. The EDIs of all four toxic elements were calculated and compared with international guidelines. Hazard Quotients for all elements were also calculated. We conclude that neither EDIs nor HQs were exceeded the WHO guidelines. Consumption of selected staple foods based on investigated toxic elements may pose no risk to human health. However, further studies are needed using more foods samples and the source of high Pb concentration in chicken needs to be further investigated.

## Figures and Tables

**Table 1:** Quality control of measurement for toxic elements (As, Cd, Pb and Cr) among four types of food: rice, wheat, red meat and chicken.

Toxic Element	As	Cd	Pb	Cr
QC (%) *	94.10	98.20	97.35	94.15
Recovery (%) ^$^				
Rice	92.52	109.67	115.63	96.14
Wheat	90.59	108.80	113.27	91.85
Meat	94.18	109.77	115.28	90.14
Chicken	93.16	106.65	112.25	92.65

* Mixed concentration (20 μg/L) of each element was measured after every five measurements; for purpose of continuing calibration verification (CCV). ^$^ Sample of each food type was spiked by adding a mixture of concentration (20 μg/L) of each element.

**Table 2:** Ranges and mean concentrations (mg/kg) of toxic elements (As, Cd, Pb and Cr) measured in 25 samples of the four food types (rice, wheat, red meat and chicken) in wet weight samples: rice (*n* = 8), wheat (*n* = 5), meat (*n* = 4) and chicken (*n* = 8).

Food Type	Toxic Elements (mg/kg)			
	As	Cd	Pb	Cr
Rice	0.02–0.07 (0.03)	0.00–0.20 (0.03)	0.00–0.13 (0.04)	0.00–0.65 (0.23)
Wheat	0.00–0.003 (0.001)	0.00–0.03 (0.01)	0.00–0.31 (0.14)	0.00–0.75 (0.15)
Meat	0.01–0.01 (0.01)	0.00–0.01 (0.01)	0.01–0.03 (0.01)	0.00–0.76 (0.25)
Chicken	0.00–0.03 (0.01)	0.06–0.07 (0.06)	0.53–0.70 (0.62)	0.07–0.26 (0.14)
Total means	0.01	0.03	0.20	0.19

**Table 3:** Daily intake (DI) of the toxic elements (As, Cd, Pb and Cr) in 25 samples of the four food types (rice, wheat, red meat and chicken).

DI of the Toxic Elements (µg/day)				
Food Type	As	Cd	Pb	Cr
Rice	8.18	8.24	9.36	56.93
Wheat	0.08	1.50	23.11	24.24
Meat	1.86	1.01	1.62	50.71
Chicken	1.24	15.37	149.79	34.03
Total	11.36	26.12	183.88	165.91

**Table 4:** The PTWI of toxic elements (As, Cd, Pb and Cr) and their PMTDI.

No.	Elements	PTWI	PMTDI	Reference
1	As	3.0 μg/kg bw per week	0.42 μg/kg bw per day	[25]
2	Cd	7 μg/Kg bw per week	1 μg/kg bw per day	[26]
3	Pb	25 μg/kg bw per week	3.57 μg/kg bw per day	[27]
4	Cr	0.7 mg/kg bw per week	0.1 mg/kg bw per day *	[28]

* Calculated value based on PTWI.

**Table 5:** Estimated dietary intake (EDI) of the toxic elements (As, Cd, Pb and Cr) in 25 samples of the four food types (Rice, wheat, red meat and chicken), the Hazard Quotient (HQ) was also reported.

EDI of the Toxic Elements (As, Cd, Pb and Cr) (mg/kg bw day)				
Food Type	As	Cd	Pb	Cr
Rice	1.14 × 10^−4^	1.15 × 10^−4^	1.31 × 10^−4^	7.96 × 10^−^^4^
Wheat	1.11 × 10^−6^	2.10 × 10^−5^	3.23 × 10^−4^	3.39 × 10^−4^
Meat	2.60 × 10^−5^	1.42 × 10^−5^	2.26 × 10^−5^	7.09 × 10^−4^
Chicken	1.73 × 10^−5^	2.15 × 10^−4^	2.09 × 10^−3^	4.76 × 10^−4^
Hazard Quotient (HQ)				
Rice	0.27	0.12	0.04	0.01
Wheat	2.64 × 10^−3^	2.1 × 10^−2^	9.1 × 10^−2^	3.4 × 10^−3^
Meat	0.06	0.01	0.01	0.01
Chicken	0.04	0.21	0.59	4.76 × 10^−3^
